# Association Between Infectious Agents and Lesions in Post-Weaned Piglets and Fattening Heavy Pigs With Porcine Respiratory Disease Complex (PRDC)

**DOI:** 10.3389/fvets.2020.00636

**Published:** 2020-09-11

**Authors:** Jessica Ruggeri, Cristian Salogni, Stefano Giovannini, Nicoletta Vitale, Maria Beatrice Boniotti, Attilio Corradi, Paolo Pozzi, Paolo Pasquali, Giovanni Loris Alborali

**Affiliations:** ^1^Istituto Zooprofilattico Sperimentale della Lombardia e dell'Emilia Romagna [Experimental Zooprophylactic Institute of Lombardia and Emilia Romagna], Brescia, Italy; ^2^Department of Veterinary Sciences, University of Parma, Parma, Italy; ^3^Department of Veterinary Sciences, University of Torino, Turin, Italy; ^4^Department of Food Safety, Nutrition and Veterinary Public Health, Istituto Superiore di Sanità, Rome, Italy

**Keywords:** porcine respiratory disease complex (PRDC), pig, lung lesion, multifactorial disease, diagnostic protocol

## Abstract

Porcine Respiratory Disease Complex (PRDC) is a multifactorial syndrome that causes health problems in growing pigs and economic losses to farmers. The etiological factors involved can be bacteria, viruses, or mycoplasmas. However, environmental stressors associated with farm management can influence the status of the animal's health. The role and impact of different microorganisms in the development of the disease can be complex, and these are not fully understood. The severity of lesions are a consequence of synergism and combination of different factors. The aim of this study was to systematically analyse samples, conferred to the Veterinary Diagnostic Laboratory (IZSLER, Brescia), with a standardized diagnostic protocol in case of suspected PRDC. During necropsy, the lungs and carcasses were analyzed to determine the severity and extension of lesions. Gross lung lesions were classified according to a pre-established scheme adapted from literature. Furthermore, pulmonary, pleural, and nasal lesions were scored to determine their severity and extension. Finally, the presence of infectious agents was investigated to identify the microorganisms involved in the cases studied. During the years 2014–2016, 1,658 samples of lungs and carcasses with PRDC from 863 farms were analyzed; among them 931 and 727 samples were from weaned piglets and fattening pigs, respectively. The most frequently observed lesions were characteristic of catarrhal bronchopneumonia, broncho-interstitial pneumonia, pleuropneumonia, and pleuritis. Some pathogens identified were correlated to specific lesions, whereas other pathogens to various lesions. These underline the need for the establishment of control and treatment programmes for individual farms.

## Introduction

Porcine Respiratory Disease Complex (PRDC) is a multifactorial disease that affects growing pigs in different stages of production, causing economic losses. This complex syndrome is influenced by the presence of several types of pathogens (porcine reproductive and respiratory syndrome virus (PRRSV), porcine circovirus (PCV)-type 2, *Mycoplasma hyopneumoniae, Mycoplasma hyorhinis, Pasteurella multocida, Haemophilus parasuis*, etc.), along with environmental conditions, as temperature, dust, ammonia, carbon dioxide and airborne bacteria and farm management (1–4).

PRDC is a major burden in piggeries worldwide because of the consequent economic losses. In affected farms, considerable costs are associated with high percentages of mortality (2–20%) and morbidity (10–40%), therapy, and limited growth performances (5, 6). Reportedly, infection with *M. hyopneumoniae* causes major economic losses to the pig industry, mainly because of reduced performance, uneven growth, increase in the number of days to reach slaughter weight, treatment and control, and increase in mortality rate when complicated infections occur (7). However, the economic impact of *M. hyopneumoniae* subclinical infection was inferred only once based on the difference in average daily weight gain (ADWG) (38 g/day) between seropositive and seronegative pigs from 18 different cohorts (8). Various studies have demonstrated the economic impact of lung lesions on growth performances. The results were mostly based on the relationship between lung lesions observed at the slaughterhouse and ADWG (9). Some authors have reported a reduction of 6–16% in the growth rate of finishing pigs (10, 11). The main study on respiratory diseases in Italian piggeries started with the observation of lung and pleural lesions (and correspondent scores) while slaughtering. The results demonstrated that diseases affecting the respiratory tract greatly prevail, and they are very likely underestimated in live animals (12, 13). Inspection while slaughtering is a valid tool to estimate the incidence of PRDC in pigs. The most commonly recorded lesion corresponds to catarrhal bronchopneumonia mainly affecting cranial lobes. This is frequently associated with interstitial pneumonia and pleuritis (14). In Italy, the percentage of catarrhal bronchopneumonia associated with enzootic pneumonia is 46.4% and that of pleuritis is 47.5% (13). These lesions indicate the evolution or exacerbation of respiratory diseases affecting pigs during the farrow-finishing period.

On the contrary, the evaluation of lesions in piglets or pigs during PRDC outbreaks is a valid tool to estimate the type of acute lesions, their extension, and the possible involvement of serosa and nasal mucosa. Furthermore, the isolation of etiological pathogens is easier in acute lesions than in chronic ones. As a consequence, treatment or the control of each outbreak should be addressed according to the specific farm situation to limit the generic use of antimicrobials.

The aim of this study was to investigate the association between lesions and infectious agents and to assess the association between nasal, pleural, and lung scores, in order to gain insights about the etiological agents associated with PRDC. The novelty of this study is to assess the etiology and lesions in samples from dead pigs with clinical suspect of respiratory disease.

## Materials and Methods

### Samples

A standardized diagnostic protocol was applied to growing pigs that died because of respiratory diseases, conferred to the Veterinary Diagnostic Laboratory (IZSLER, Brescia), during 2014–2016. Table 1 shows the total number and the production stage of the sampled pigs. A further distinction per year was made to carry out a temporal assessment.

**Table 1:** Data on samples analyzed during 2014–2016, presented as number of animals, number of investigated cases, and production stage.

**Year**	**N**°** of animals**	**N**°** of investigated cases**	**Weaned piglets**	**Growing/fattening pigs**
2014	510	257	295 (57.8%)	215 (42.2%)
2015	622	320	334 (53.7%)	288 (46.3%)
2016	524	286	301 (57.5%)	223 (42.5%)
Total	1,656	863	930 (56.2%)	726 (43.8%)

The protocol used for the qualitative and quantitative evaluation of the lung, pleura, and nasal lesions is associated with a systematic monitoring of pathogens. It was applied to carcasses or organs (lungs) submitted to the Diagnostic Laboratory of Istituto Zooprofilattico Sperimentale della Lombardia e dell'Emilia-Romagna with the suspicion of respiratory disease, which was then confirmed during necropsy.

Lung, pleural, and nasal lesion scores were registered during necropsy with other information such as that on the productive stage and laboratory investigations performed.

### Classification of Lung Lesions

The scheme for the classification of lesions was adapted from published methods (15).

The lesions of the organs were identified to be associated with the following diseases/conditions: catarrhal bronchopneumonia (CBP), purulent bronchopneumonia (PBP), interstitial pneumonia (IP), interstitial bronchopneumonia (BIP), pleuropneumonia (PP), pleuritis (PL), pericarditis (PE), and pleuro-pericarditis (PL-PE).

CBP is characterized by lesions of parenchyma and bronchi affecting principally the cranial, cardiac, and anterior portions of diaphragmatic lobes. It is characterized by mucus and catarrhal exudate in the lumen of the bronchus tree, by parenchymal consolidation and by interstitial space thickening. The appearance of the lungs varies from red to light brown and changes to a grayish color during chronic infection.

PBP is characterized by lesions of lung parenchyma and bronchi, which are characterized by mucus and catarrhal-purulent exudate in the lumen of the bronchus tree. Generally, it is a totally disseminated complication of CPB or BIP. Hence, cellular detritus and stagnated exudates favor the replication of pyogenic bacteria. These lesions develop abscess formations detectable with palpation.

IP is characterized by serosal exudation into alveolar walls and by interstitial oedema. Parenchyma suffers from incremented consistency, and the color changes from light red to purple red during the acute phase and to light pink during the chronic phase. These lesions are commonly associated with viral infections and involve the entire affected lung. Unfortunately, this lesion can be masked (by other lesions) or be complicated, and it can evolve into broncho-interstitial pneumonia. BIP is a complication of IP because it also involves the bronchus tree. In particular, serosal exudation is found in the lumen of bronchi and bronchioles.

PP is a fibrinous/necrotising pneumonia associated with pleuritis and affects the dorso-caudal portions of the diaphragmatic lobes. A hyperacute lesion is characterized by an increased consistency and by a color variation of the parenchyma that ranges from brown to red. An acute lesion is characterized by an increased consistency of the affected portions of parenchyma and by an alternation of the red area with a lighter pink area, which is a result of fibrin deposition. The chronic evolution is characterized by abscess formations and sequestrum, complicated by fibrous adherences between lung lesions and chest wall.

PL, PE, and PL-PE are characterized by inflammation of the pleura and pericardium (or both) and by fibrinous exudation that can evolve in adherences.

### Lung, Pleural, and Nasal Scores

Here, irrespectively. to the character of the lung lesion (as above assessed), we scored (0–4 points) the extension of the lesion in each lobe, according to Madec and Derrien (16) and Madec and Kobish (17). SPES (Slaughterhouse Pleuritis Evaluation System) was applied to score (0–4 points) pleural lesions (18). Nasal lesions were scored on a 6-point scale (0–5 points) according to the system described by de Jong (19).

### Laboratory Investigations

The lung, heart, pericardium, lymph nodes, pericardial and pleural fluids, and tracheobronchial swabs were processed to conduct, histology, microbiological examinations, and molecular identification.

For bacteriological examination, we inoculated the processed samples into blood agar and Gassner agar (Reparto Produzione Terreni – IZSLER, Brescia) and incubated the culture plates for 24–48 h at 37°C and 5% CO_2_ to identify *Actinobacillus pleuropneumoniae, P. multocida, Streptococcus* spp., and *Actinomyces pyogenes*, the bacterial cultures were subjected to Gram's staining and confirmation with biochemical identification and serotyping. Furthermore, to isolate NAD-dependent pathogens (*A. pleuropneumoniae* and *H. parasuis*), blood agar cultures were cross-streaked with a *Staphylococcus intermedius* to evaluate colony-satellitism. Furthermore, DNA extraction was applied to pure cultures of *A. pleuropneumoniae* in order to serotype each isolated strain by an end-point PCR, as described below.

Viral isolation of SIV (Swine Influenza Virus) was performed. A lung fragment was homogenized with Minimum Essential Medium Eagle (Sigma-Aldrich) containing Streptomycin, Penicillin G, and Sulfate Streptomycin. The solution was centrifuged at 1,500 rpm for 5 min. An aliquot of supernatant was used to infect cells (MDBK or Caco-2).

Molecular identification of PRRSV, PCV-type 2, *M. hyopneumoniae, M. hyorhinis, H. parasuis*, and *A. pleuropneumoniae* was performed.

Several commercial kits were used for Nucleic Acid Extraction: NucleoMag Vet 200 (Macherey-Nagel) for PRRSV and PCV-type 2; Rneasy mini kit (Qiagen) for SIV; Dneasy Blood & Tissue kit (Qiagen) for *M. hyopneumoniae, M. hyorhinis*, and *H. parasuis*. Finally, DNA boiling extraction was applied to a pure cultures of *A. pleuropneumoniae* (98°C for 10 min with 1,050 rpm oscillation).

An end-point PCR was performed to confirm the presence of *H. parasuis* according to the protocol described by Oliveira et al. (20), and *A. pleuropneumoniae* serotyping was performed according to the protocol described by Xie et al. (21).

A Real Time RT-PCR for PRRSV detection from blood and lung tissue homogenate was performed using the^1^ following the manufacturer's instructions.

A Real Time PCR for PCV-type2 detection from lung tissue homogenate and inguinal lymph node homogenate was performed in accordance with the protocol described by Olvera et al. (22).

A Real Time RT-PCR for SIV detection was performed from lung tissue homogenate in accordance with the protocol described by Spackman et al. (23).

A Real Time PCR for *M. hyopneumoniae* detection was performed from lung tissue homogenate in accordance with the protocol described by Marois et al. (24).

A Real Time PCR for *M. hyorhinis* detection was performed from lung tissue homogenate in accordance with the protocol described by Tocqueville et al. (25).

Finally, a Real Time PCR for *A. pleuropneumoniae* detection from trachea-bronchial swabs and lung tissue homogenate was performed in accordance with the protocol described by Tobias et al. (26).

### Statistical Analysis

The proportion of each type of lesion was calculated, and the binomial exact method was used to compute 95% confidence intervals (95% CI). The association of the production stage and pathological lesions with pathogens was assessed by Fisher's exact test (FET). For *A. pyogenes, A. pleuropneumoniae, Streptococcus spp*., and PCV-type 2, multivariate logistic regression models were employed, with pathogens as dependent variables and production stage and pathological lesions as covariate. To identify a possible association among different scores and to a verify score distribution, Spearman's correlation coefficient (r) was calculated between different scores (lung vs. pleural scores, lung vs. nasal scores, and pleural vs. nasal scores). Furthermore, a Chi-square test (3 × 2) was performed to compare the scores according to the different classes. The classes identified were: slight (0–9), moderate (10–19), and severe (20–28) for the lung score; slight (0–2) and severe (3, 4) for the pleural score; and slight (0–2) and severe (3–5) for the nasal score. The Chi-square test (2 × 2) was also performed to compare pleural and nasal scores. For each test a *p* < 0.05 was considered statistically significant. All analyses were performed using R software (R version 3.3.1, R Core Team, R Foundation for Statistical Computing. R: A language and environment for statistical computing, http://www.R-project.org/; 2016 [accessed 31/05/2017]).

## Results

The proportions of weaned piglets and growing/fattening pigs with PRDC were 57.8 and 42.2%, respectively, in 2014; 53.7 and 46.3%, respectively, in 2015; and 57.5 and 42.5%, respectively, in 2016 (Table 1). Four hundred sixty-one farms conferred pigs 863 times for diagnostic investigations, with a cumulative number of 1,656 pigs. The mean number of pigs conferred for each farm was 3.6 (SD 4.4) and the mean of times each farm conferred pigs was 1.9 (SD 1.9).

### Pleuritis Is the Most Frequently Observed Lesion in Both Groups of Animals Affected by PRDC

**Figure 1:**
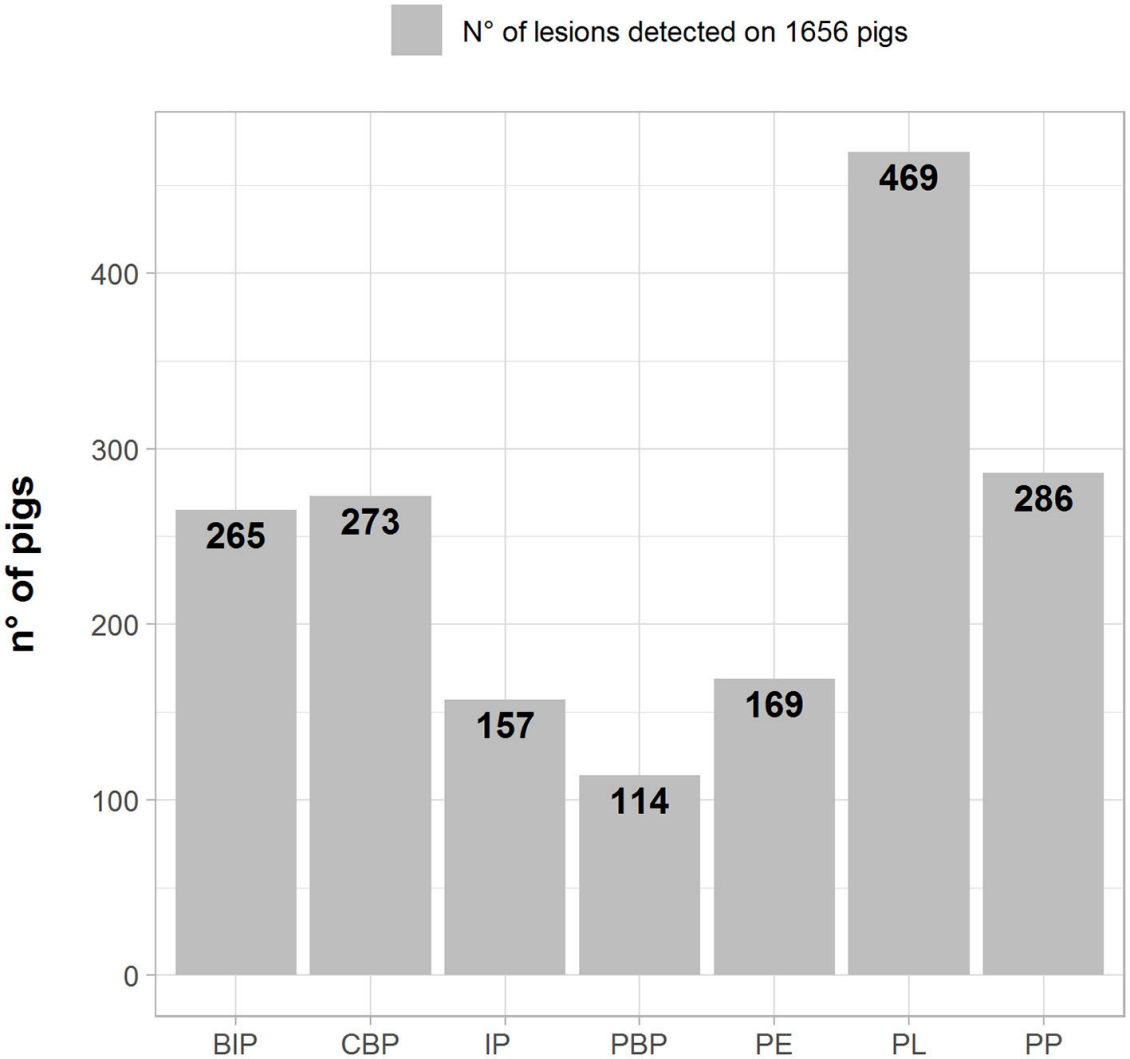
Number (left axes/gray bars) and percentage (right axes/black lines) of different pathological lesions observed in BIP, Broncho-Interstitial Pneumonia; CBP, Catarrhal Bronchopneumonia; IP, Interstitial Pneumonia; PBP, Purulent Bronchopneumonia; PE, Pericarditis; PL, Pleuritis; and PP, Pleuropneumonia.

The frequencies of lesion distribution in the lungs, pleura, and pericardium are depicted in Figure 1 (number of pigs with lesions). PL was recorded in the highest number of samples (469; 28.3%), followed by pleuropneumonia (PP) (286; 17.2%), catarrhal bronchopneumonia (CBP) (273; 16.5%), and BIP (265; 16.0%).

The frequencies of lesion distributions in the organs of weaned piglets and growing/fattening pigs are depicted in Table 2. PL was the most frequent lesion observed in both the groups (253 and 216 cases, respectively). Other frequent lesions corresponded to CBP (184), BIP (122), PP (125), PE (119), and PL-PE (77) in weaned piglets. On the contrary, PP and BIP were frequently observed in fattening pigs (161 and 143, respectively). The association between the production stage and pathological lesions was statistically significant for IP, PP, PBP, PL, and PE. The post-weaning group showed a lower probability of developing pathological lesions associated with PP, PBP, and PE and a higher probability to developing pathological lesions associated with IP and PL than did the growing group.

**Table 2:** Number, percentage (%), and 95% CI of pathological lesions detected in pigs according to production stage.

**Pathological**	***N***°****	**Growing/fattening**			**Post-weaning**			***p*-value**	**OR**	**OR CI 95%**
**Lesions**		***N***°****	**%**	**CI95%**	***N***°****	**%**	**CI 95%**			
CBP	273	89	32.6	27–38.1	184	67	61.8–72.9	0.0000	1.76	1.33–2.35
IP	157	83	52.8	45–60.6	74	47	39.3–54.9	0.0179	0.67	0.47–0.94
BIP	265	143	53.9	47.9–59.9	122	46	40–52	0.0003	0.62	0.47–0.81
PP	286	161	56.2	50.5–62	125	44	37.9–49.4	0.0000	0.55	0.42–0.71
PBP	114	59	51.7	42.5–60.9	55	48	39–57.4	0.0792		
PL	469	216	46	41.5–50.5	253	54	49.4–58.4	0.2717		
PE	169	50	29.5	22.7–36.4	119	70	63.5–77.2	0.0001	1.98	1.39–2.86

*The association between the pathological lesions and the production stage was calculated by Fisher's exact test; when the association was statistically significant (p < 0.05), the odds ratio (OR) was calculated using the growing group as the source of baseline data*.

### Detection of Respiratory Pathogens

The proportions of respiratory pathogens in the samples collected from weaned piglets or fattening pigs are depicted in Table 3. The association between the production stage and the isolated pathogen was statistically significant for *Streptococcus suis, P. multocida, A. pleuropneumoniae*, PRRSV, PCV-type 2, *M. hyopneumoniae, M. hyorhinis*, and *H. parasuis*. The post-weaning group was more likely to show *S. suis*, PRRSV, *M. hyorhinis*, and *H. parasuis* than was the growing group. *M. hyorhinis* was the most commonly detected pathogen in the lungs of weaned piglets with lesions (98.7%). In the lungs of weaned piglets with and without lesions, PRRSV (75 and 59.6%), *H. parasuis* (61.3 and 38.2%), and *Streptococcus* spp. (45.3 and 34.8%) were the most commonly detected pathogens.

**Table 3:** Pathogens detected, proportion and 95% CI according to production stage.

**Respiratory pathogen**	**N**°** isolated**	**Growing/fattening**			**Post-weaning**			***p*-value**	**OR**	**OR CI 95%**
		***N***°****	**%**	**CI 95%**	***N***°****	**%**	**CI 95%**			
*Streptococcus* spp	372	160	43	38.0–48.0	212	57	52.0–62.0	0.722		
*Streptococcus suis*	17	3	18	0.5–35.8	14	82	64.2–100	0.046	3.68	1.02–20.05
*A. pyogenes*	72	31	43	31.6–54.5	41	57	45.5–68.4	0.904		
*P. multocida*	213	109	51	44.5–57.9	104	49	42.1–55.5	0.021	0.71	0.53–0.96
*A. pleuropneumoniae*	264	167	63	57.4–69.1	97	37	30.9–42.6	0.001	0.39	0.29–0.52
*PRRS*	946	344	36	33.3–39.4	602	64	60.6–66.7	0.001	2.24	1.79–2.80
*PCV-type2*	455	251	55	50.6–59.7	204	45	40.3–49.4	0.001	0.47	0.37–0.60
SIV	192	94	49	41.9-56.0	98	51	44.0-58.1	0.16		
*M. hyopneumoniae*	141	99	70	62.7–77.8	42	30	22.2–37.3	0.001	0.15	0.09–0.25
*M. hyorhinis*	217	43	20	14.5–25.1	174	80	74.9-85.5	0.001	23.85	6.54–131.94
*H. parasuis*	185	58	31	24.7–38.0	127	69	62.0–75.3	0.016	1.75	1.09–2.82

*The association between the pathogens isolated and the production stage was calculated using Fisher's exact test; when the association was statistically significant (p < 0.05), OR was calculated using the growing group as the source of baseline data*.

Differently, the growing group was more likely to show *P. multocida, A. pleuropneumoniae*, PCV-type 2, and *M. hyopneumoniae*.

*M. hyopneumoniae* (68.5%) and PCV-type 2 (43.7%) were the most commonly detected pathogens in the organs of fattening pigs with lesions, whereas, in the organs of fattening pigs with and without lesions, PRRSV (55.1 and 41.3%), *Streptococcus* spp. (41 and 18.7%), and *P. multocida* (26.3 and 14.7%) were the most frequently detected pathogens.

### Distribution Between a Single Lesion and Respiratory Pathogen Detection in Weaned Piglets and Fattening Pigs

**Figure 2:**
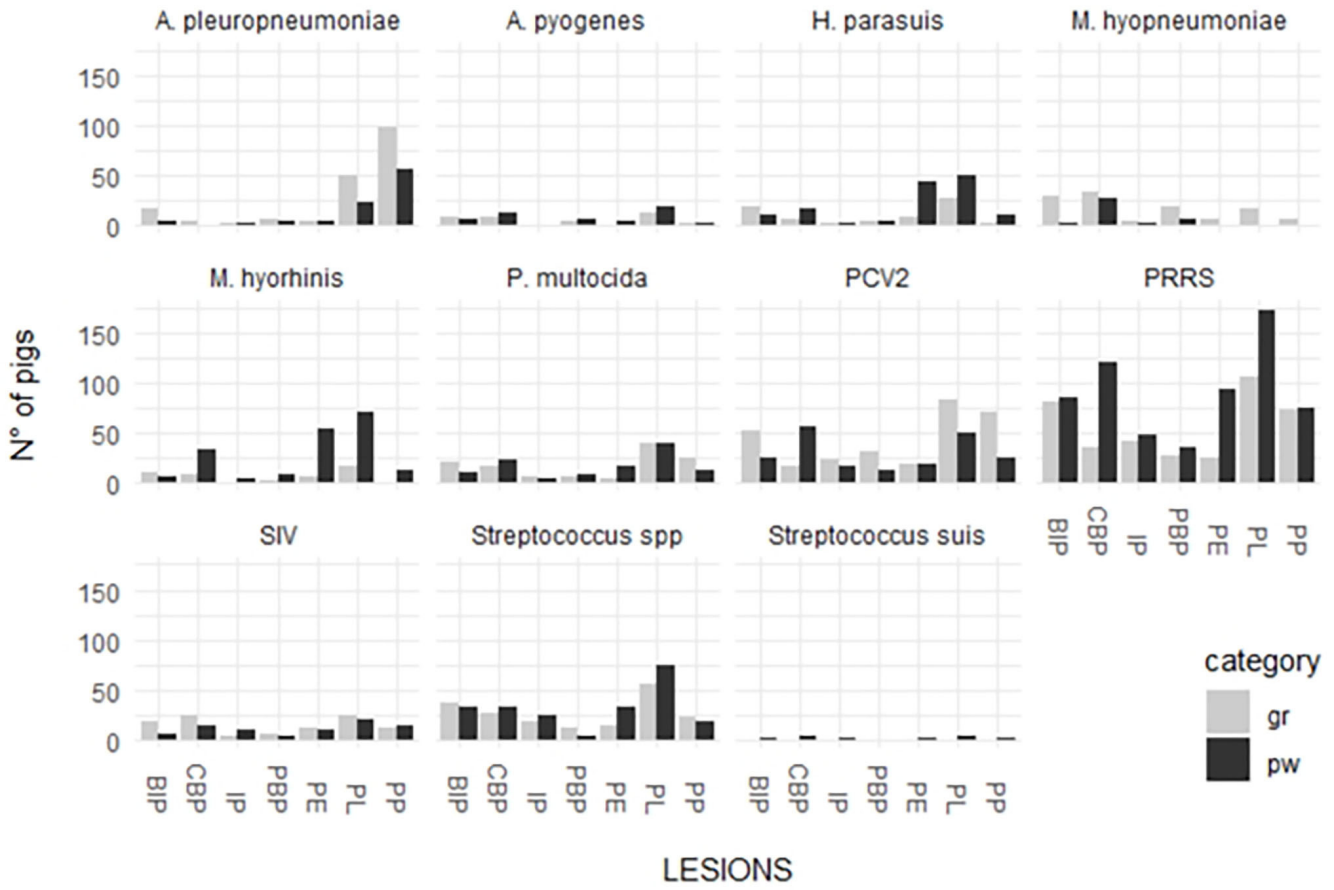
Distribution of detected respiratory pathogens in lesions recorded in weaned piglets and fattening pigs.

The distribution between pathogen detection and each single lesion in samples collected from weaned piglets and fattening pigs are depicted in Figure 2.

*P. multocida* was detected in the organs of weaned piglets affected by the lesions associated with the following diseases/conditions: PBP (28.6%); PL (26%); CBP (25.9%); and PE (25%). This pathogen was detected in different proportions in the organs of fattening pigs with the lesions associated with the following diseases/conditions: CBP (37.8%); PL (29.9%); BIP (28%); PBP (28%); and IP (23.3%). *A pleuropneumoniae* was detected in the organs of weaned piglets affected by the lesions associated with the following diseases/conditions: PP (64.4%); PL (16.7%); and PBP (14.3%). This pathogen was detected in different proportions in the organs of fattening pigs with the lesions associated with the following diseases/conditions: PP (81%); PL (38.10%); PBP (28%); BIP (24%); and PE (20.8%).

*H. parasuis* was detected in the organs of weaned piglets affected by the lesions associated with the following diseases/conditions: BIP (76.9%); PP (73.3%); PE (70.4%); and PL (60.7%). This pathogen was detected in different proportions in the organs of fattening pigs with the lesions associated with the following diseases/conditions: PBP (66.3%); CBP (58.3%); BIP (52.8%); IP (50%); PL (50%); PE (50%); and PP (25%).

*Streptococcus* spp. was detected in the organs of weaned piglets affected by the lesions associated with the following diseases/conditions: IP (83%); BIP (59%); PL (61%); and PE (50%). The pathogen was also detected in high proportions in the organs of fattening pigs with the lesions associated with the following diseases/conditions: IP (63%); CBP (60%); PBP (52%); BIP (51%); PE (63%); and PL (57%).

*A. pyogenes* was detected in the organs of both groups affected by purulent bronchopneumonia (21% in weaned piglets and 20% in fattening pigs). The pathogen was also detected in the organs with the lesions associated with the following diseases/conditions: CBP (14% in weaned piglets and 18% in fattening pigs); BIP (12% in weaned piglets and 11% in fattening pigs); and PL (12% in weaned piglets and 10% in fattening pigs).

*M hyorhinis* was detected in high proportions in the organs of weaned piglets (percentage range 70–90%) and in low proportions in the lungs affected by BIP (53.8%). The pathogen was detected in low proportions in the organs of fattening pigs showing lesions associated with the following diseases/conditions: CBP (66.7%); PBP (60%); PL-PE (85.7%); and PE (38.9%).

*M. hyopneumoniae* was scarcely detected in the lung and serosal lesions of weaned piglets (38.9% in PBP and 30.9% in CBP). On the contrary, it was detected in high proportions in the lung and serosal lesions of fattening pigs (percentage range 65–90%) with the exception of those associated with IP (44.4%) and pleuropneumonia.

PRRSV was highly associated with lung and serosal lesions of both the groups. The detection percentages of different lesions were higher in weaned piglets than in fattening pigs. The rate of detection in weaned piglets varied from 65.8% (PP) to 84.7% (PE) and that in fattening pigs varied from 45.6% (CBP) to 66.7% (PL). The highest percentages of detection were associated with PL (66.7%) and BIP (62.5%).

SIV was associated with PL in weaned piglets (30.8%) and CBP (30.4%), PE (26.1%), and PL (33.3%) in fattening pigs.

PCV-type 2 was more associated with lung and serosal lesions in fattening pigs than with those in weaned piglets. The rate of detection in weaned piglets varied from 17.6% (PE) to 37.3% (CBP) and that in fattening pigs varied from 25.4% (CBP) to 65.3% (PBP).

As reported in Table 4, *A. pyogenes* was associated with the following: CBP (FET: *p* < 0.05; OR, 2.02; 95% CI, 1.12–3.52), IP (FET: *p* < 0.05; OR, 0.13; 95% CI, 0.003–0.754), PP (FET: *p* < 0.05; OR, 0.35; 95% CI, 0.11–0.86), PBP (FET: *p* < 0.05; OR, 2.6; 95% CI, 1.20–5.17), and PL (FET: *p* < 0.001; OR, 2.1; 95% CI, 1.3–3.5). The detection of *A. pyogenes* made the detection of PP and IP lesions twice less probable and CBP, PBL, and PL lesions it twice more probable.

**Table 4:** Number of pathogens isolated from pathological lesions detected in weaned piglets and fattening pigs.

**Pathogens**	**Isolated**	**CBP**	**IP**	**BIP**	**PP**	**PBP**	**PL**	**PE**
*Streptococcus spp*.	372	58	44	72	41^*^^I^	18	129^*^^I^	49^*^^I^
*Streptococcus suis*	17	4	1	1	1	0	4	3
*A. pyogenes*	72	20^*^^I^	1^*^^D^	15	5^*^^D^	11^*^^I^	32^*^^I^	4
*P. multocida*	213	39	11^*^^D^	31	39	15	79^*^^I^	21
*A. pleuropneumoniae*	264	5^*^^D^	4^*^^D^	23^*^^D^	153^*^^I^	11	74	10^*^^D^
*PRRSV*	946	157	89	165	147	62	279	118^*^^I^
*PCV-type2*	455	73	38	78	94^*^^I^	45^*^^I^	132	38^*^^D^
*SIV*	192	39	14	24	26	12	46	23
*M. hyopneumoniae*	141	62	6	32	7	26^*^^I^	18	6
*M. hyorhinis*	217	41	4	17^*^^D^	13	11	86	60
*H. parasuis*	185	23	4	29	13	8	79	52

*The association between the detected pathogen (yes/no) and pathological lesions (yes/no) was calculated by Fisher exact test*.

* * Statistically significant association. I = presence of pathogens increased the probability of detecting a lesion. D = the presence of pathogens decreased the probability of detecting a lesion*.

*A. pleuropneumoniae* was associated with the following: CBP (FET: *p* < 0.0001; OR, 0.08; 95% CI, 0.03–0.2), IP (FET: *p* < 0.0001; OR, 0.13; 95% CI, 0.03–0.33), BIP (FET: *p* < 0.0001; OR, 0.45; 95% CI, 0.28–0.72), PP (FET: *p* < 0.0001; OR, 13.0; 95% CI, 9.53–17.85), and PE (FET: *p* < 0.0001; OR, 0.31; 95% CI, 1.20–5.17). The detection of *A. pleuropneumoniae* made the detection of CBP, IP, BIP, and PE lesions 13 times less probable and that of PP lesions 13 times more probable.

*M. hyopneumoniae* was associated with PBP (FET: *p* < 0.001; OR, 2.91; 95% CI, 1.38–6.45). The detection of *M. hyopneumoniae* doubled the probability of detection of PBP lesions.

*M. hyorhinis* was associated with BIP (FET: *p* < 0.0001; OR, 0.14; 95% CI, 0.05–0.45). The detection of *M. hyorhinis* decreased the probability of detection of BIP lesions.

*P. multocida* was associated with IP (FET: *p* < 0.05; OR, 0.49; 95% CI, 0.23–0.91) and PL (FET: *p* < 0.01; OR, 1.59; 95% CI, 1.16–2.17). The detection of *P. multocida* decreased the probability of detection of IP lesions and nearly doubled the probability of detection of PL lesions.

PCV-type 2 was associated with pathological lesions of PP (FET: *p* < 0.01; OR, 1.47; 95% CI, 1.08–1.99), PBP (FET: *p* < 0.001; OR, 1.92; 95% CI, 1.23–3.01), and PE (FET: *p* < 0.05; OR, 0.65; 95% CI, 0.43–0.96). The detection of PCV-type 2 decreased the probability of detection of PE pathological lesions and increased the probability of detection of PP and PBP pathological lesions.

PRRSV was associated with PE pathological lesions (FET: *p* < 0.001; OR, 1.84; 95% CI, 1.24–2.76). The detection of PRRSV nearly doubled the probability of detection of BIP lesions.

*Streptococcus* spp. was associated with pathological lesions of PP (FET: *p* < 0.0001; OR, 0.52; 95% CI, 0.36–0.75), PL (FET: *p* < 0.001; OR, 1.47; 95% CI, 1.14–1.90), and PE (FET: *p* < 0.05; OR, 1.47; 95% CI, 1.01–2.12). The detection of *Streptococcus* spp. decreased the probability of detection of PP lesions and increased the probability of detection of PL and PE lesions by one and an half times (Table 4).

### Multivariate Analysis

Multivariate regression logistic models were applied, with pathogens (*A. pyogenes, A. pleuropneumoniae, Streptococcus spp.*, and PCV-type 2) as dependent variables and production stage and pathological lesions as covariates. The results of the multivariate regression analysis are shown in Table 5. For *A. pyogenes*, the best model (LRT, 33.99; df = 3; *p* < 0.0001) included CBP, PBP, and PL lesions. The detection of *A. pyogenes* was four times more probable with CBP, PBL, and PL lesions.

**Table 5:** Factors that were found to be statistically significant by multivariate regression analysis for *A. pyogenes, A. pleuropneumoniae, Streptococcus* spp., and PCV-type 2 pathogens.

**Pathogens**	**Factor**	**Baseline**	**OR**	**OR 95% CI**		***p*-value**
*A. pyogenes*	CBP	Lesion detected	4.40	2.27	8.61	<0.0001
	PBP	Lesion detected	3.77	1.77	7.46	<0.0001
	PL	Lesion detected	3.55	2.01	6.44	<0.0001
*A. pleuropneumoniae*	CBP	Lesion not detected	7.61	3.36	21.89	<0.0001
	IP	Lesion not detected	6.62	2.67	21.95	<0.0001
	BIP	Lesion not detected	1.70	1.05	2.85	0.03
	PP	Lesion detected	7.21	5.16	10.14	<0.0001
	PE	Lesion not detected	2.51	1.33	5.28	<0.0001
	Production stage	Growing	2.27	1.66	3.10	<0.0001
*PCV-type2*	PBP	Lesion detected	1.82	1.18	2.80	0.001
	Production stage	Growing	2.08	1.65	2.62	<0.0001
*Streptococcus spp*.	PP	Lesion not detected	1.90	1.35	2.74	0.001

*OR and 95% CI were calculated by multivariate logistic regression*.

For *A. pleuropneumoniae*, the best model (*p* < 0.0001) included all factors that were found to be statistically significant by FET, as well as production stage. The presence of *A. pleuropneumoniae* was more likely to occur in the growing group than in the post-weaning group with PP lesions and without CBP, IP, BIP, and PE lesions.

For PCV-type 2, the best model (LRT, 48.3; df = 2; *p* < 0.0001) included PCP lesion and production stage. The presence of PCV-type2 was more likely to occur in the growing group than in the post-weaning group with PCP lesions.

Finally, for *Streptococcus* spp. the best model (LRT, 14.3; df = 1; *p* < 0.0001) included PP lesion. The presence of *Streptococcus* spp. was more likely to occur in the absence of PP lesions.

### Isolation of Single Pathogens in Organs Without Lesions

Among 1,656 lung samples, 234 non-lesioned samples were observed (14.1%, 95% CI, 12.5–15.9%); in particular, among these 234 samples, 161 (68.8%, 95% CI, 62.4–74.7%) and 73 (31.2%, 95% CI, 25.3–37.6%) were identified in the post-weaning and growing groups. The association between the production stage and the lesions was statistically significant (FET: *p* < 0.0001), with the post-weaning group showing a lower probability of presenting lesions (OR, 0.53; 95% CI, 0.39–0.73). Table 6 shows the frequencies and percentages of the pathogens detected in the lungs without lesions according to the production stage. The *p*-value obtained by FET indicates the association between the pathogen and the production stage. Statistically significant associations of the production stage were found with *A. pleuropneumoniae* (FET: *p* = 0.017), *M. hyopneumoniae* (FET: *p* = 0.049), and *M. hyorhinis* (FET: *p* < 0.001). The post-weaning group showed lower probabilities of detection of *A. pleuropneumoniae* (OR, 0.3; 95% CI, 0.1–0.8) and *M. hyopneumoniae* (OR, 0.1; 95% CI, 0.1–0.9) than did the growing group. A different pattern was observed for *M. hyorhinis*; it was mostly detected in the post-weaning group (OR, 3.14; 95% CI, 4.4–∞).

**Table 6:** Frequency (n) and percentage (%) of detection of respiratory pathogens in samples without lesions are shown at global level (overall) and by production stage (growing/post-weaning).

**Pathogen**	**Overall**			**Growing**		**Post-weaning**		***p*-value**
	**Without lesions**	**Positive**	**%**	***n***	**%**	***n***	**%**	
*Streptococcus* spp.	234	46	19.7%	11	23.9%	35	76.1%	0.288
*Streptococcus suis*	234	6	2.6%	3	50.0%	3	50.0%	0.379
*A. pyogenes*	234	4	1.7%	2	50.0%	2	50.0%	0.591
*P. multocida*	234	20	8.5%	6	30.0%	14	70.0%	1.000
***A. pleuropneumoniae***	**234**	**19**	**8.1%**	**11**	**57.9%**	**8**	**42.1%**	**0.017**
PRRSV	204	133	65.2%	35	26.3%	98	73.7%	0.058
*PCV-type2*	185	45	24.3%	15	33.3%	30	66.7%	0.572
SIV	202	40	19.8%	12	30.0%	28	70.0%	1.000
**M**. ***hyopneumoniae***	**18**	**7**	**38.9%**	**6**	**85.7%**	**1**	**14.3%**	**0.049**
**M**. ***hyorhinis***	**56**	**51**	**91.1%**	**7**	**13.7%**	**44**	**86.3%**	**0.000**
*H. parasuis*	66	41	62.1%	7	17.1%	34	82.9%	0.535

*p-value was calculated using Fisher's exact test to assess the association between the occurrence of pathogens and the production stage*.

### Relation Between Nasal, Pleural, and Lung Scores

The mean lung score was 13.53 in weaned piglets and 12.14 in fattening pigs With 14.7% of the samples being non-lesioned (equaly distributed between weaned and fattening pigs). The mean pleural score was 1.46 and 1.48, in the weaned and fattening pigs, respectively, with 42.71 and 39.10% of the samples, respectively, being non-lesioned. The mean nasal score was 0.50 and 0.78 in the weaned and fattening pigs, respectively, with 61.82 and 42.71% of the samples, respectively, being non-lesioned.

**Figure 3:**
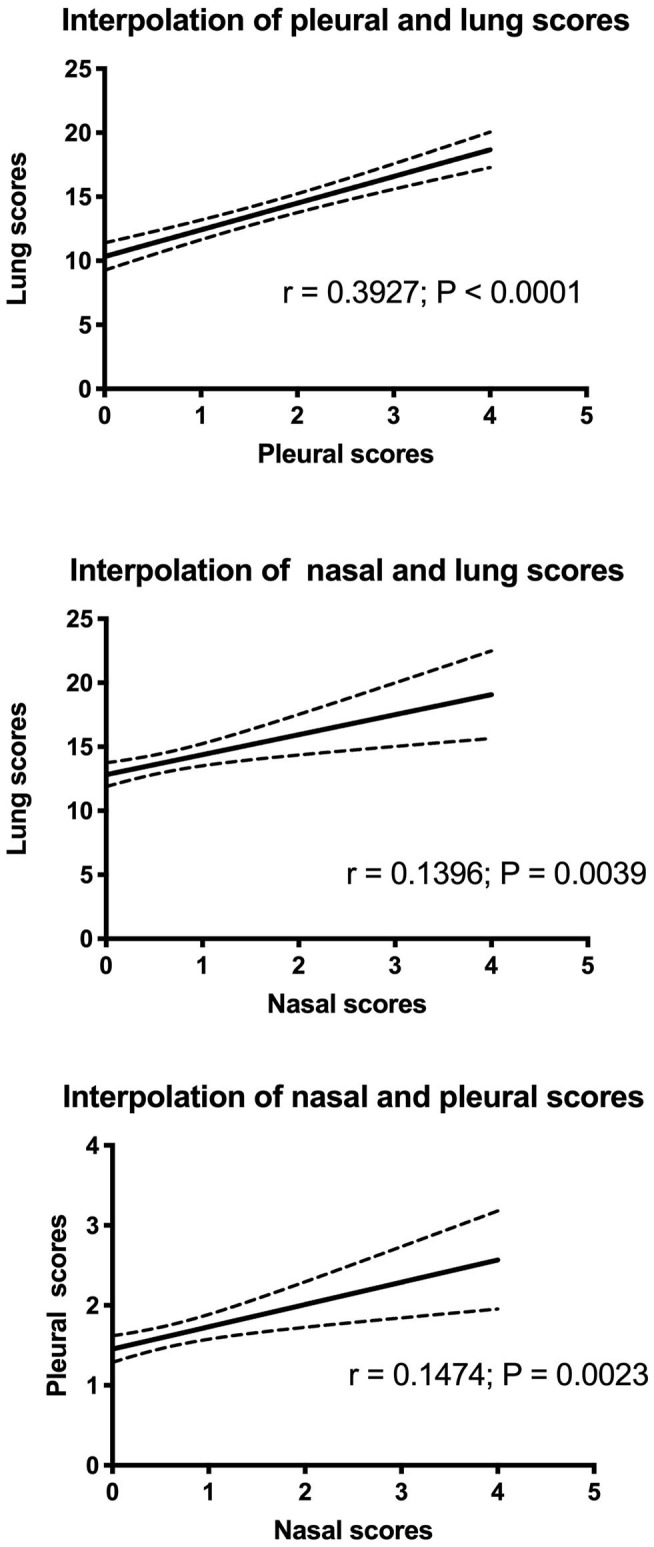
Interpolation of lung, pleural, and nasal scores.

Data distributions and correlations between the different scores are shown in Figure 3. The results indicate that the difference was statistically significant among scores (*p* < 0.0001, lung score vs. pleural score; *p* = 0.0039, lung score vs. nasal score; *p* = 0.0023, pleural score vs. nasal score). However, the Spearman r value was low, indicating a discrete/limited correlation (*r* = 0.39; *r* = 0.14; *r* = 0.15, respectively). On the contrary, Chi-square test showed that there was a statistically significant relationship between lung and pleural scores (3 × 2; *p* < 0.0001) and that there was no statistically significant relationship between the lung and nasal scores (3 × 2) and between the pleural and nasal scores (2 × 2); however, *p* was 0.0572 in the case relationship between the lung and nasal scores (Supplementary Material).

## Discussion

The systematic application of the standardized protocol during 2014–2016 allowed the identification of the principal lesions and pathogens associated with PRDC outbreaks in growing pigs and the comparison of different situations. Our results indicate that the PRDC percentages recorded during the 3 years of study were almost constant between the weaned and the fattening pigs and that PRDC principally affects the weaned pigs (53–58 vs. 42–47%) causing high mortality. The proportion of lung lesions was high with values comparable to those reported by previous investigations at slaughterhouses (12, 13, 27–29). Nevertheless, it is important to highlight that the frequency of pleuritis, BIP, and pleuropneumonia along with that of IP and purulent bronchopneumonia was similar between piglets and pigs. Conversely, catarrhal bronchopneumonia, pericarditis, and pleuro-pericarditis affected principally weaned piglets. Generally, pleuritis lesions were more frequently detected in weaned piglets. The comparison between these results and the data obtained from slaughtered pigs with catarrhal bronchopneumonia and pleuropneumonia (12, 13), highlighted an evolution of the lesions from the growing stage to the slaughtering stage.

The analysis of the association between lesions and the pathogens involved showed that the presence of *A. pleuropneumoniae* is largely associated with pleuropneumonia in fattening pigs. *A. pleuropneumoniae* is the agent associated with porcine pleuropneumonia, a contagious respiratory disease capable of causing significant economic losses to the swine industry worldwide (30).

Frequently, pigs that overcome acute diseases remain chronically infected, showing no clinical signs, but likely harboring chronic lung alterations, such as fibroblastic pleurisy and lung tissue sequesters surrounded by fibrotic tissue (13, 31). In this study, in fact, *A. pleuropneumoniae* was strongly associated with lesions characteristic of pleuropneumonia with a minor involvement of pleura. In addition, we isolated *A. pleuropneumoniae* from non-lesioned lungs in 8.1% of the cases; this finding suggests that *A. pleuropneumoniae* is unlikely to be associated with a subclinical lung condition.

*Streptococcus* spp. and *S. suis* were associated with pleural and pericardial lesions, mainly in post-weaning pigs. Fibrinous or fibrinopurulent pleuritis, peritonitis, or polyserositis were identified in pigs infected with both *S. suis* and *H. parasuis*. Pigs in which only *S. suis* was isolated had a more extensive suppurative exudation than that was associated with *H. parasuis. S. suis* in pigs with pleuritis, peritonitis, and polyserositis should be considered first during differential diagnosis, especially when the exudate was more suppurative than fibrinous (32). In this study, the frequency of *Streptococcus* spp. and *S. suis* detection in non-lesioned lungs was 19.7 and 2.6%, respectively. This finding is partly in contrast with other published data, which showed that asymptomatic colonization of *S. suis* in the upper respiratory tract as well as in the intestinal and genital tract was common (33).

*M. hyopneumoniae* was significantly more prevalent in cases of purulent bronchopneumoniae and even more apparently prevalent in catarrhal bronchopneumoniae. The lesions were more frequent in fattening pigs than in weaned piglets. *M. hyopneumoniae* is an important respiratory pathogen capable of causing the disease by itself or in combination with other pathogens (34, 35). A combination was observed between *M. hyopneumoniae* and PRRSV or *S*. suis (36, 37). Different studies have demonstrated that disease severity in growing pigs is correlated with several factors (38, 39). The fact that *M. hyopneumoniae* was frequently found in organs without lesions corroborates the finding that it is a common pathogen with effects that are likely dependent on other triggering factors, however we cannot exclude that *M. hyopneumoniae* has been detected in the first stage of the infection.

The correlation between lung and pleural scores was found to be linear with *p* < 0.0001. However, it was a moderate correlation because Spearman's r-coefficient was low (*r* < 0.4). Probably, this aspect is associated with a wide distribution of lung scores in each class of pleural scores. For this reason, a further comparison between scores was performed using the Chi-squared test. Lung scores were divided into three categories, while pleural scores were divided into two categories. The results indicated that the frequency of severe lesions in lungs corresponded to a high frequency of severe pleural lesions. Therefore, the results suggest that the proposed scoring approach could be a reliable system to evaluate lesions in the respiratory tracts during pathological investigations.

In conclusion, these data shed light on the impact of different pathogens on the respiratory disease of pigs and highlight the need for a systematic diagnostic approach to manage the respiratory disease in pig farms.

## Data Availability Statement

The raw data supporting the conclusions of this article will be made available by the authors, without undue reservation.

## Ethics Statement

Animal approval was not required according to national/local legislation as we exclusively analyzed the remains of pigs that died naturally. Written informed consent was obtained from the owners of the animals studied.

## Author Contributions

JR, PPa, and GA contributed to the conception and design of the study, acquisition of data, analysis and interpretation of data, and drafting of the article. CS, SG, and NV contributed to the acquisition of data, analysis and interpretation of data, and drafting of the article. BB contributed to the acquisition of data, analysis and interpretation of data, and revise revisions of the article. AC and PPo contributed to the analysis and interpretation of data and revision of the article. All authors contributed to the revision of the manuscript and furthermore they read and approved the submitted version.

## Conflict of Interest

The authors declare that the research was conducted in the absence of any commercial or financial relationships that could be construed as a potential conflict of interest.

## Footnotes

^1^LSI VetMAX (TM) PRRSV EU/NA Real-Time PCR kit (Thermo Fisher).

## Abbreviations

PRDC: Porcine Respiratory Disease Complex
PRRSV: Porcine Reproductive and Respiratory Syndrome Virus
PCV: type 2: Porcine Circovirus Type 2
ADWG: Average Daily Weight Gain
CBP: Catarrhal Bronchopneumonia
PBP: Purulent Bronchopneumonia
IP: Interstitial Pneumonia
BIP: Interstitial Bronchopneumonia
PP: Pleuropneumonia
PL: Pleuritis
PE: Pericarditis
PL-PE: Pleuro-pericarditis
SD: standard deviation
SPES: Slaughterhouse Pleuritis Evaluation System
SIV: Swine Influenza Virus
PCR: Polymerase Chain Reaction
CI: Confidence Interval
OR: Odds Ratio.
