# Supplementary material for: Association Between Infectious Agents and Lesions in Post-Weaned Piglets and Fattening Heavy Pigs With Porcine Respiratory Disease Complex (PRDC)
Source: Front Vet Sci. 2020 Sep 11;7:636. doi: 10.3389/fvets.2020.00636 (PMC7516008; doi:10.3389/fvets.2020.00636)

**Supplementary data**. Frequency distribution of samples according to lung and pleura scores (A); lung and nasal scores (B) and nasal and pleura scores (C). Numbers refers to the cumulative numbers of samples with the respective scores.

A


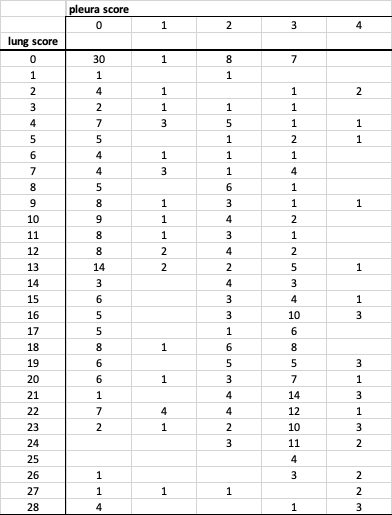


B


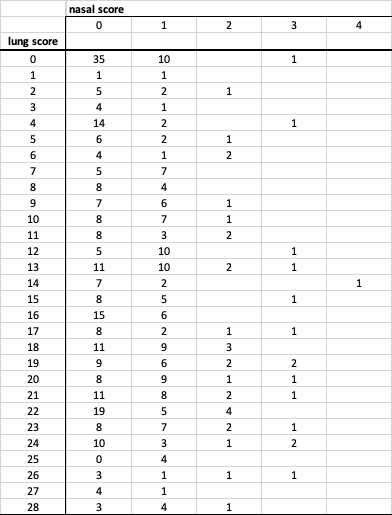


C


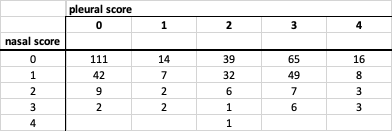

Supplement: Supplementary file 1 [file Table_1.DOCX]
